# Self-touch: Contact durations and point of touch of spontaneous facial self-touches differ depending on cognitive and emotional load

**DOI:** 10.1371/journal.pone.0213677

**Published:** 2019-03-12

**Authors:** Stephanie Margarete Mueller, Sven Martin, Martin Grunwald

**Affiliations:** Haptic Research Lab, Paul Flechsig Institute of Brain Research, University of Leipzig, Leipzig, Germany; Liverpool Hope University, UNITED KINGDOM

## Abstract

Every human being spontaneously touches its eyes, cheeks, chin and mouth manifold every day. These spontaneous facial self-touches (sFST) are elicited with little or no awareness and are distinct from gestures and instrumental acts. Self-touch frequency has been shown to be influenced by negative affect and attention distraction and may be involved in regulating emotion and working memory functions. Yet, even though self-touch research dates back several decades fundamental aspects, like the temporal progression of sFST or the effects of executing hand and touched face area, have not yet been analyzed. For the first time, the present study measured sFST temporal aspects to the millisecond using accelerometers and EMG. Spontaneous self-touch was triggered in sixty participants who completed a delayed memory task of complex haptic relief stimuli while listening to distracting aversive sounds. We found that while both hands were used equally often and with the same overall movement times and contact durations, significant effects occurred for face area in both frequency and contact durations. Ergo the point of touch seems to have some relevance of its own, independently of which hand is used to perform it. The results show that not only frequency but also the point of touch and contact durations are influenced by cognitive and emotional demands. We argue that investigating the fundamental characteristics of sFST will further the understanding of cognitive focusing and attentional mechanisms.

## Introduction

Self-touches, just like posture shifts, object manipulations and mannerisms, are types of self-regulatory movements and have been discussed to be associated with psychological and cognitive processes [1–6]. Self-touch movements are usually performed with one or both hands and involve rubbing, scratching, caressing, or grooming any part of the body’s surface or pieces of clothing or accessories [4,7].

Self-touches are performed manifold every day by every human being. These movements are not usually designed to communicate and are frequently accomplished with little or no awareness [8,7]. They are distinct from gestures and instrumental acts in so far as they are not related to speech content or task-oriented manipulations, respectively [4,7,9].

Even though self-touches (ST) may seem to appear in response to itching skin sensations or grooming needs, many research results imply associations of ST with underlying negative affect, anxiety or discomfort and have been discussed to serve to comfort and release emotional arousal [8,10,4,11,6].

ST have also been interpreted as manifestations of distraction susceptibility and attentional focusing and the organism’s attempts to shield itself from distractions [12,2,13].

So far, possible bio-psychological functions of ST have mostly been discussed on the basis of behavioral data. Therefore, it remained largely unclear whether the ST movements merely represent energetic discharges and ‘emotional leakages’ [8] or if they actually have an effect on cortical functioning. The, as of yet, only existing neurophysiological study of spontaneous self-touch behavior gave first indications of the brain regulatory functions of ST [6]. The authors analyzed the cortical power changes before and after facial self-touch during a memory task. Analyses revealed significant changes in EEG theta power above central and parietal brain regions shortly before and after facial ST. However, when the participants were asked to touch their face in a similar manner, no EEG effects were found. These findings gave the first neuro-physiological support to the assumed emotion and working memory regulating functions of spontaneous ST [6].

### Contact duration & movement time

However, much about ST remains unknown. So far, most studies have focused on changes in ST frequency. Little is known about the temporal aspects of ST.

Freedman and his colleagues differentiated between discrete and continuous forms of self-touching [14,15,7]. Discrete self-touches were described as brief (3 seconds or less) and non-continuous, like a stroke of the chin or touching an eye, and mainly directed to the face or the head [4,16,14]. Continuous body-focused movements lasted substantially longer (in some instances more than 100 sec.) and were further subdivided into 3 groups: continuous stimulation of the hands by each other (hand-to-hand movements), the body-surface by one or two hands (continuous direct) and continuous manipulations of articles of clothing or accessories (continuous indirect) [14].

Studies that have counted the abundance of ST types and their localization showed that the majority of ST are directed towards the face and the hands [4,17]. Even though discrete facial self-touches have been described as the most frequent of human self-touch behaviors [4,17] they have rarely been analyzed in detail. If they are counted separately at all, they are often categorized as fidgeting or grooming behaviors [18] or are discussed together with the effects of continuous self-touches [11,14,19,20]. Ekman & Friesen (1972) mentioned an increase of facial ST (while the occurrence of other forms of ST remained the same) in subjects who had to tell deceptive stories compared to honest ones [10].

However, in the above mentioned neuro-physiological study of spontaneous ST behavior, Grunwald et al. (2014) analyzed the spectral power of discrete facial ST incidents [6]. These findings show, that discrete, brief facial self-touches do not merely serve to scratch an itch or display the acting person’s nervousness but may form a class of their own in the broad field of body-directed movements. The results revealed that these short face touches were associated with the restoration of cortical theta power, indicating their involvement in emotional homeostasis and working memory maintenance.

To date the movement processes and contact durations of ST have not been analyzed in detail. Previous attempts have been made via observation through a one-way mirror or by recording and evaluating videotapes (e.g. [11,14,19,20]). Both methods yield rough estimates and are likely to be skewed due to reaction time delays of the observer.

In light of the above results, the present study is designed to examine discrete facial ST in more detail.

To further the discussion about the effects of ST on human brain functioning, we want to analyze the hand preference, point of touch and the temporal aspects of sFST during a spatial memory task (replicated from [6].

### Cognitive and emotional load

Previous studies have shown increased occurrences of ST to be related to negative emotional states and stress during interpersonal situations as well as to attentional disruptions during cognitive demands [21,22]. During the present experiment subjects were required to maintain complex and extensive working memory content while listening to aversive sounds. This experimental setting has proven to facilitate the occurrence of sFST in a previous study [6]. In line with the distraction susceptibility and attentional focusing hypothesis of Barroso and his colleagues [12,2,13] we expect to observe higher numbers of sFST during the retention interval than during all other phases of the experiment (Hypothesis 1a).

Barroso and colleagues also proposed that continuous concentration may warrant longer ST [12]. During the retention interval the participants will have to keep two complex geometric forms in mind, while warding off internally appearing thoughts, memories and feelings that may distract them from the memory task. These intrusions may occur idiopathically or may be triggered by the presented sounds. Due to the continuous concentration required during RI, we expect sFST, which occur during RI, to be of longer duration than those that occur during other experimental phases (Hypothesis 1b).

**Fig 1:**
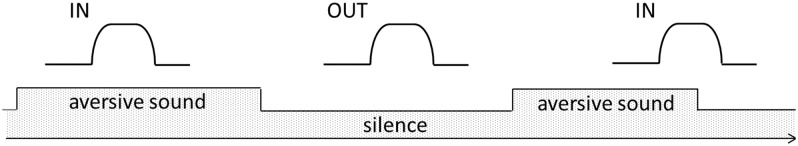
Types of spontaneous facial self-touch (sFST) during the retention interval. IN = sFST during acoustic stimulation; OUT = sFST between two sounds. Each retention interval lasted approximately 14 minutes and consisted of 40 sounds and 40 silences. Duration of the sounds and silences varied between 7 and 13 seconds to prevent habituation and anticipation effects.

During RI the participants will try to keep the memory of the two relief structures actively alive on their visuospatial scratchpad [23]. We assume that the sounds will distract the subjects’ concentration from their working memory load and focus their attention on the occurring sounds much like an orienting reflex. Various researches of auditory distraction and attention capture have shown that changing-state acoustic stimuli are especially potent in capturing attention when the memory load is of serial nature and sounds are unpredictable [24–27]. In the present study 60 different sounds were used which occurred at random and were separated by silences of different lengths. According to the above cited literature these changing-state sounds are especially hard to ignore. Each new sound captures the attention anew and distracts the participant’s concentration from the memory task. Brief ST may help the participants to focus their attention back on the memory task and block out the respective distracting sound. We assume brief sFST to be instantaneous responses to sudden attentional distractors as opposed to continuous ST, which seem to be associated with ongoing distracting emotional states like nervousness or social anxiety [15,14]. Since the present experiment was designed to trigger short distractions during a phase of sustained arousal while focusing on a spatial memory task, sFST should occur more often during the aversive sounds (IN) than in between them (OUT; Fig 1; Hypothesis 1c).

### Laterality

To date, no conclusive theory has tackled the problem of uni- or bilateral and ipsi- or contralateral self-touches. Several researchers have shown that participants performed ST equally often with the left and the right hand during natural speaking about self-chosen or emotionally neutral topics [28,29,18]. However, when test subjects had to solve different cognitive tasks, like making up the last line of incomplete limericks, creating a crossword puzzle, performing figure reconstruction or mental rotation, left handed self-touching movements were predominant [28,3]. In another condition the test subjects were asked to solve a nonverbal visual task of finding and outlining a hidden figure. During this experiment significantly more right-handed ST occurred [28]. Furthermore, studies on patients with depressive symptoms have shown a higher incidence in left-handed ST (both discrete and continuous) during clinical interviews [30]. When the symptoms improved the significant difference in laterality subsided [30]. Similarly, persons with strong field dependence showed a significant asymmetry of continuous ST of the left hand acting on the right hand [31]. Furthermore, Ruggieri and colleagues found that when female psychology students recalled and told embarrassing personal stories left-handed ST occurred more frequently and lasted significantly longer than right-handed ST. When the same students were asked more neutral questions, about their daily routines and their last vacation, right-handed ST occurred relatively more often [32,33]. The authors attributed the increase in right-handed ST to the order of succession of their experimental questions: since the more neutral questions were asked first, the subjects may have felt initial social anxiety during the first interpersonal contact with a stranger who asks them personal questions [33].

One possible explanation for the situational variation of ST-laterality may be that the sensorimotor activation of the hand mirrors the predominant hemispheric activation of any given task or situation [32,33].

Taken together, current results suggest an association of left-handed ST with anxiety, negative emotional states and the processing of cognitive tasks while right-handed ST may be associated with cognitive uncertainty or social anxiety during interpersonal contact.

Based on these findings we expect to observe more left-handed ST in the course of the present study, since the aversive sounds during the retention interval may induce negative emotional states in the participants while the memory task should pose continuous cognitive demands (Hypothesis 2a). In line with Ruggieri et al (1982) we expect movement times and contact durations to be significantly longer for left-handed ST than for right-handed ST (Hypothesis 2b).

So far, studies investigating the laterality of ST have focused solely on the executing hand and did not consider the contacted body area. While some studies have reported differences in how frequently various body areas were touched, these findings were merely interpreted as nonverbal communication means, self-grooming or due to cultural differences [18,15,17,34]. For example during more intense emotional states, especially if speaking is required during feelings of anxiety, continuous hand-to-hand movements have been reported to increase in frequency [20]. However, another explanation is possible: If which hand is used for self-touch indicates hemispheric activation, the same could be true for which side of the body or face is touched.

Consequently, the body area where the ST is directed may have as much informational value as the hand that performs the movement.

At present it is unknown whether facial self-touches of different durations or locations have different effects or serve different purposes. In fact, it is not yet known whether the motor aspects of ST (movement of the hand or arm towards/away from the body/face) serve a functional purpose or if solely the sensory aspects (contact of the skin) are of relevance to the organism. If the arm and hand movements are of predominant relevance, we should observe no differences between the face areas. However, we expect the actual skin contact to be the relevant aspect of ST. If the skin contact is of central relevance in the sFST process, this should be reflected in the frequency and contact durations of the different face areas. Since it is yet unknown, whether the hand movement or the skin contact serves the dominant purpose, we wanted to ascertain if either side of the face was touched with different frequencies, movement times or contact durations (Hypothesis 3a) and whether differences exist between ipsi- and contralateral ST (Hypothesis 3b).

## Methods

The data for the present study were gathered in an experiment investigating facial self-touch movements (sFST) during a delayed memory task of complex haptic stimuli (sunken reliefs [35–37]. The experimental setting has been successfully used before to induce ST [6]. The study was approved by the Ethics Committee of University of Leipzig Medical Faculty. All participants gave written informed consent.

**Fig 2:**
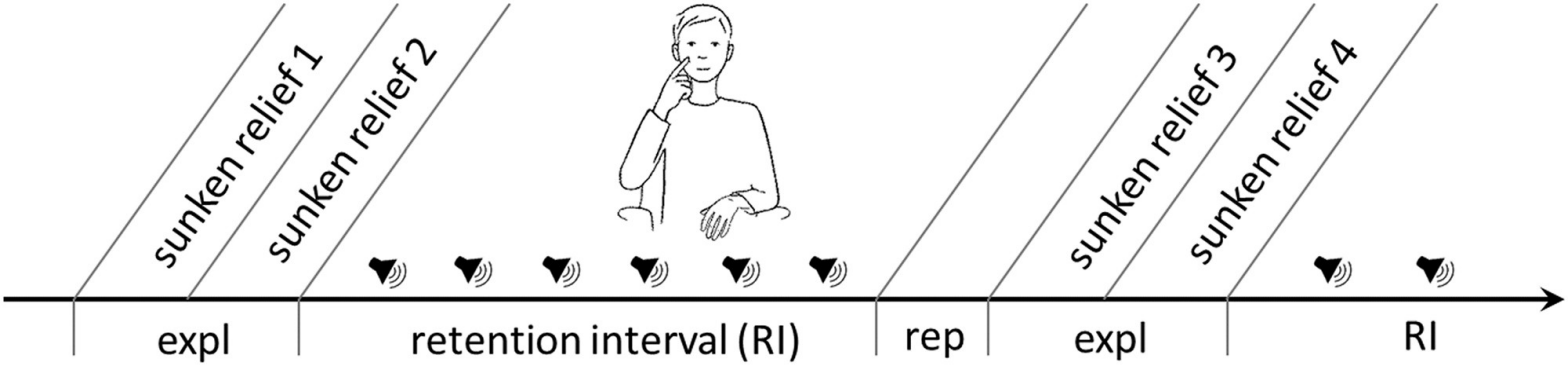
Schematic representation of the course of the experiment. To trigger spontaneous facial self-touch participants had to manually explore (expl) and subsequently remember two relief stimuli for a retention interval (RI) of 14minutes during which aversive sounds were played via loudspeaker. Afterwards they were asked to reproduce (rep) them on a sheet of paper. The process was performed twice with different relief stimuli.

In the course of the experiment (Fig 2) two haptic stimuli had to be explored, remembered for a retention interval of roughly 14 minutes and subsequently drawn on a piece of paper. After the first round the procedure was repeated a second time with two different haptic stimuli. During the retention intervals (RI) unpleasant sounds from the database of International Affective Digitized Sounds (IADS; [38]) and short sound-free periods alternated with one another. All in all we used 60 different sounds that were played randomly. Each RI consisted of 40 sounds and 40 sound free periods. The durations of the sounds and pauses varied between 7 and 13 seconds to prevent habituation and anticipation effects.

**Fig 3:**
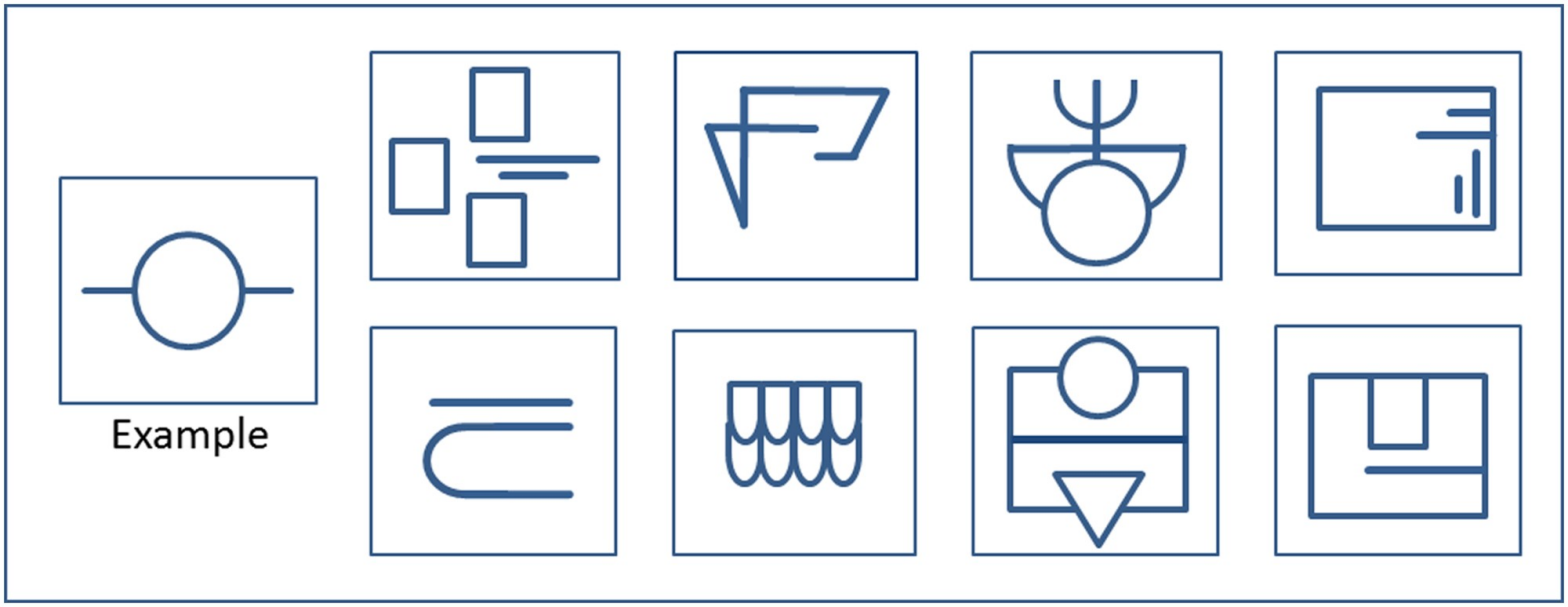
Sunken relief stimuli and example stimulus. The participants practiced manual exploration on the example stimulus. Each participant was randomly assigned 4 of the above pictured relief stimuli to be explored during the experiment.

Participants were seated in a comfortable armchair with the holding equipment (for the haptic relief stimuli) in front of them. Before the experiment began, the procedure was explained to the participants and an example stimulus as well as some example sounds were presented. When the participant had no more questions, the experiment proper began. An opaque screen obscured the participant’s hands from vision during exploration. Subjects were allowed to explore the reliefs as long as they pleased; with one or both hands. A schematic graph of the sunken reliefs is displayed in Fig 3. Each sunken relief was milled into a plastic plate of 13 x 13 cm. After exploration the opaque screen was removed so the participants could move freely without any obstructions during RI. The order of the sunken reliefs was randomized between subjects.

Facial self-touch movements and contact durations were measured via EMG (two electrodes placed on the dorsal sides of both the left and right forearm above m. extensor carpi ulnaris) and analogous, tri-axial acceleration sensors (ADXL335; attached to the wrists of the participants). The whole experiment was videotaped through an one-way mirror. The recording system (IT-med GmbH, Germany) allowed for parallel, synchronized recordings of EMG, acceleration sensors and videos of the whole experimental session with a recording rate of 256 data points per second. EEG was also recorded but the results will be presented elsewhere.

### Data analysis

The present study was designed to particularly trigger discrete, brief self-touches of the face. To define the type of sFST even more strictly, all self-touches of the hair, head, neck and ears were excluded as well as all sFST with obvious instrumental value (yawning, scratching, nose picking etc.).

**Fig 4:**
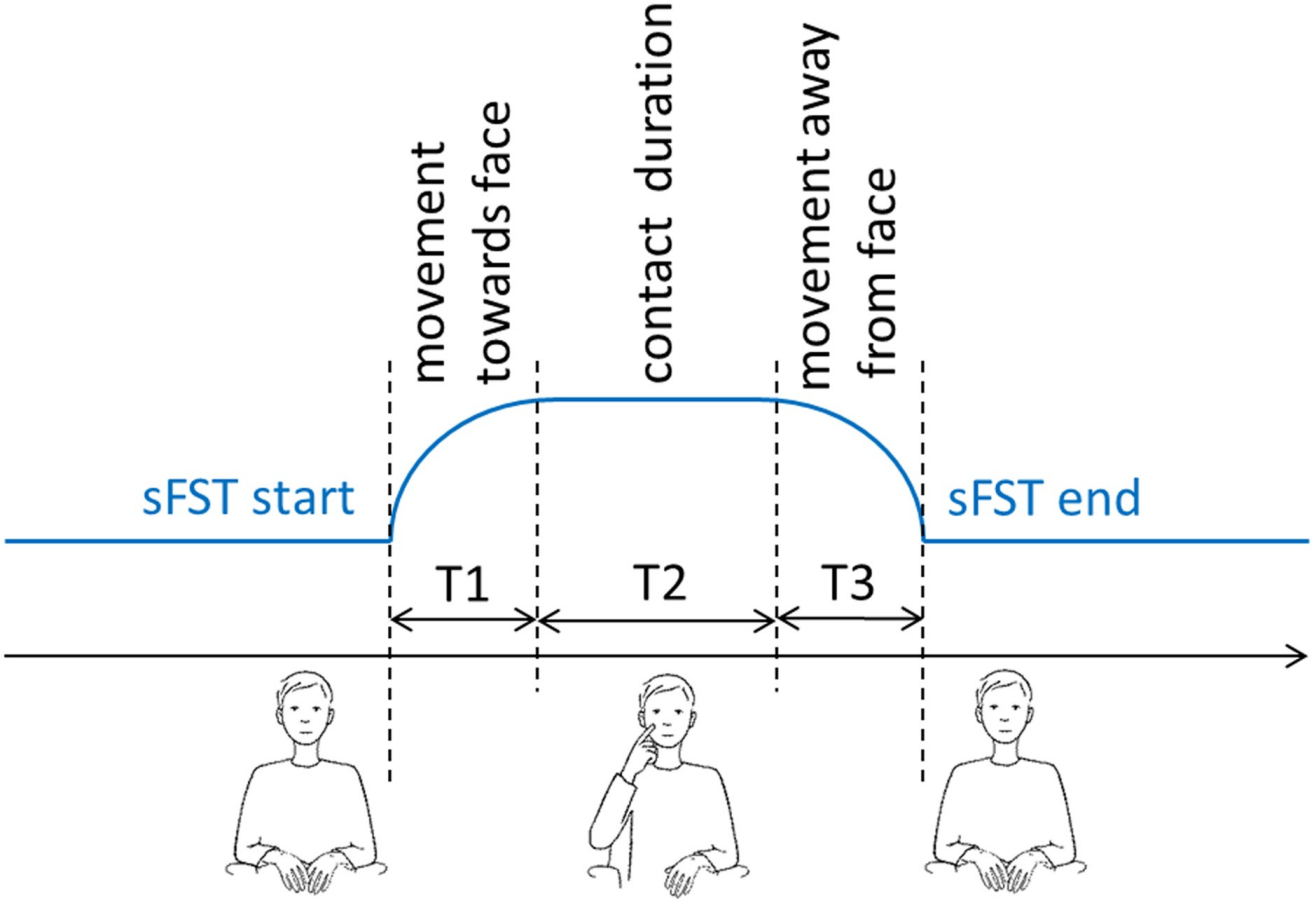
Schematic representation of the temporal structure of spontaneous facial self-touches (sFST). Illustrated by C. Maiwald.

Duration of sFST movement towards the face (T1) was defined from the beginning of muscle contraction in the lifted forearm till skin contact between finger and face (T2). Duration of ST movement away from the face (T3) was defined from the moment of loss of skin adhesion until the hand returned to a motionless resting position (Fig 4). Only those sFST with movement and contact durations shorter than 10 seconds were included in the analyses.

Even though sFST occurred during all experimental phases, the main analytical emphasis will be on sFST that occurred during the retention interval. The number of self-touches was not sufficient to conduct within-subjects comparisons for other phases of the experiment. However, where appropriate, exploratory group differences for sFST of other experimental phases were reported.

All statistical analyses were conducted using SPSS for Windows (version 24.0). Alpha was set at 5%.

Independent samples t-Test was used for independent group comparisons. For comparisons of group frequencies Binomial-Tests were conducted. Within-subjects analyses were performed via Paired-samples t-Tests and repeated-measures ANOVA for two or more measures, respectively.

The data of the current study are available from the corresponding author upon request.

### Participants

Sixty volunteers took part in the experiment (30 male; age: M = 25.72 years, SD = 3.05; age range 20–35 years). All test subjects were right-handed according to a test of handedness [39]. To prevent interfering cognitions about self-touch movements, all participants were told a cover story that the study was focused on investigating haptic memory processes. After data acquisition all participants were debriefed and received 10€/h. All test subjects took part voluntarily and signed a consent form. The study was approved by the local ethics committee.

## Results

### Descriptive statistics

Across the whole experiment 54 of the 60 participants performed on average M = 5.61 (SD = 4.81) sFST. The mean contact duration of sFST (T2) was M = 1.76 seconds (SD = 1.53), with movement times of approximately one second from the start of the movement to contact (T1: M = 0.91; SD = 0.35) and from cessation of contact till termination of hand movement (T3: M = 0.98; SD = 0.59). A subgroup of n = 45 participants (25 men/ 20 women) showed self-touch movements during the retention interval.

### Cognitive load and distraction effects

#### Hypothesis 1a—Cognitive load and sFST frequency

As expected, across all participants, significantly more sFST were performed during the retention interval (sum = 194) than during all other experimental phases combined (sum = 109; Binomial Test p < .001).

#### Hypothesis 1b—Cognitive load and temporal aspects of sFST

**Fig 5:**
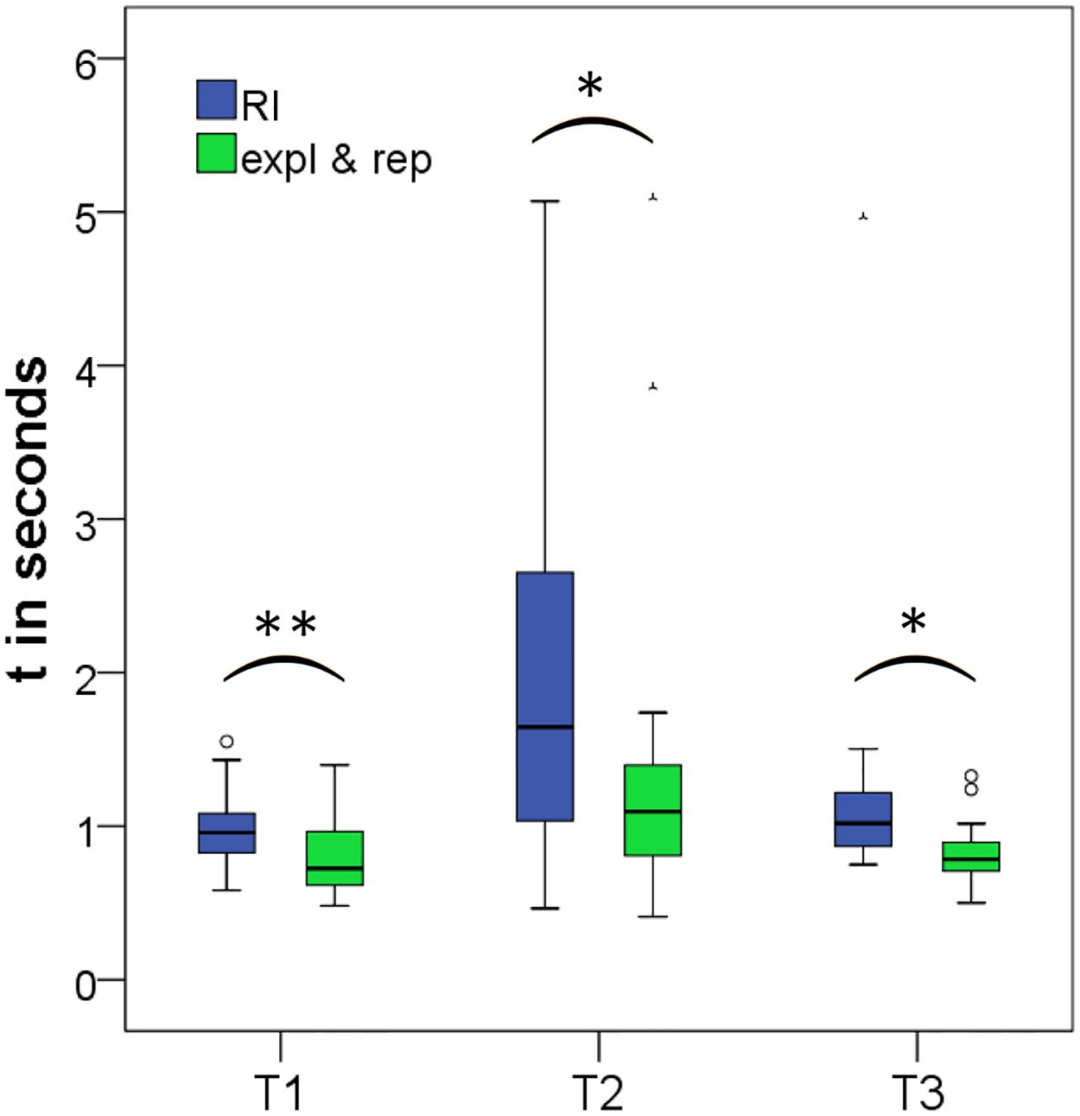
Movement times and contact durations during RI and other experimental phases (within-subjects). T1 = movement time towards face; T2 = contact duration; T3 = movement time away from face. Paired-samples t-tests revealed longer movement times and contact durations of spontaneous facial self-touch that occurred during the retention interval (RI) than during exploration and reproduction (expl & rep). *p < .05; **p < .01.

To increase restriction on the data within-subjects comparisons were used to analyze the temporal aspects of sFST. Ergo only partcipants who performed sFST during all three experimental phases (n = 30) were included in the analyses. Within-subjects comparisons revealed that movement times were significantly slower and contact durations were significantly longer during RI than during the pooled other experimental phases (T1: t(29) = 2.878, p < .01, d = 0.53; T2: t(29) = 2.477, p < .05, d = 0.45; T3: t(29) = 2.668, p < .05, d = 0.49; Table 1 & Fig 5).

**Table 1:** Movement times and contact durations of sFST in seconds per experimental phase (within-subjects, n = 30).

T1 (movement towards face)			
	expl	RI	rep
*M*	.920	.969	.742
*N* (sFST)	33	194	76
*SD*	.408	.344	.280
Minimum	.367	.277	.340
Maximum	1.875	2.219	2.043
T2 (contact duration)			
*M*	1.391	2.002	1.327
*N* (sFST)	33	194	76
*SD*	1.198	1.694	1.021
Minimum	.281	.180	.285
Maximum	5.266	9.508	5.094
T3 (movement away from face)			
*M*	.917	1.055	.819
*N* (sFST)	33	194	76
*SD*	.269	.686	.355
Minimum	.527	.434	.332
Maximum	1.922	8.887	2.043

sFST = spontaneous facial self-touch; expl = exploration; RI = retention interval; rep = reproduction

#### Hypothesis 1c—Distraction effects of aversive sounds

Frequency: Significantly more sFST occurred during the acoustic stimuli (IN) than during the silences between the sounds (OUT). This was true 1) across all subjects who performed sFST during RI (n = 45; IN = 131, OUT = 62, Binomial Test: p < .001) and 2) for within-subject comparisons: Those subjects that performed sFST both IN and OUT (n = 25) performed significantly more sFST during sounds(IN: M = 3.92, SD = 2.69; OUT: M = 2.12, SD = 1.09; t(24) = 3.781, p < .001, d = 0.76).

Temporal aspects: Again, within-subjects comparisons were performed to analyze the temporal aspects. Participants who performed at least one sFST during sounds, between sounds and during one other experimental phase (n = 19) were included in the analyses. The respective within-subjects comparisons revealed significant differences in contact durations and movement time away from the face but not for movement towards the face (Repeated measures ANOVAs: Wilks’ Lamda; Table 2).

**Table 2:** Comparison of movement times and contact durations during sounds, between sounds and during other experimental phases.

		*M*	*SD*	*N*	*F*	*df*	*error* *df*	*p*
T1	IN	.922	.195	19	0.600	2	17	.560
	OUT	.921	.327	19				
	other	.844	.249	19				
T2	IN	1.671	.657	19	5.317	2	17	< .05
	OUT	2.435	1.619	19				
	other	1.175	.630	19				
T3	IN	1.045	.367	19	3.619	2	17	< .05
	OUT	.923	.211	19				
	other	.815	.174	19				

T1 = movement time towards face; T2 = contact duration; T3 = movement time away from face; IN = sFST during acoustic stimulation; OUT = sFST during the silences between sounds; other = sFST during other experimental phases (expl, rep)

**Fig 6:**
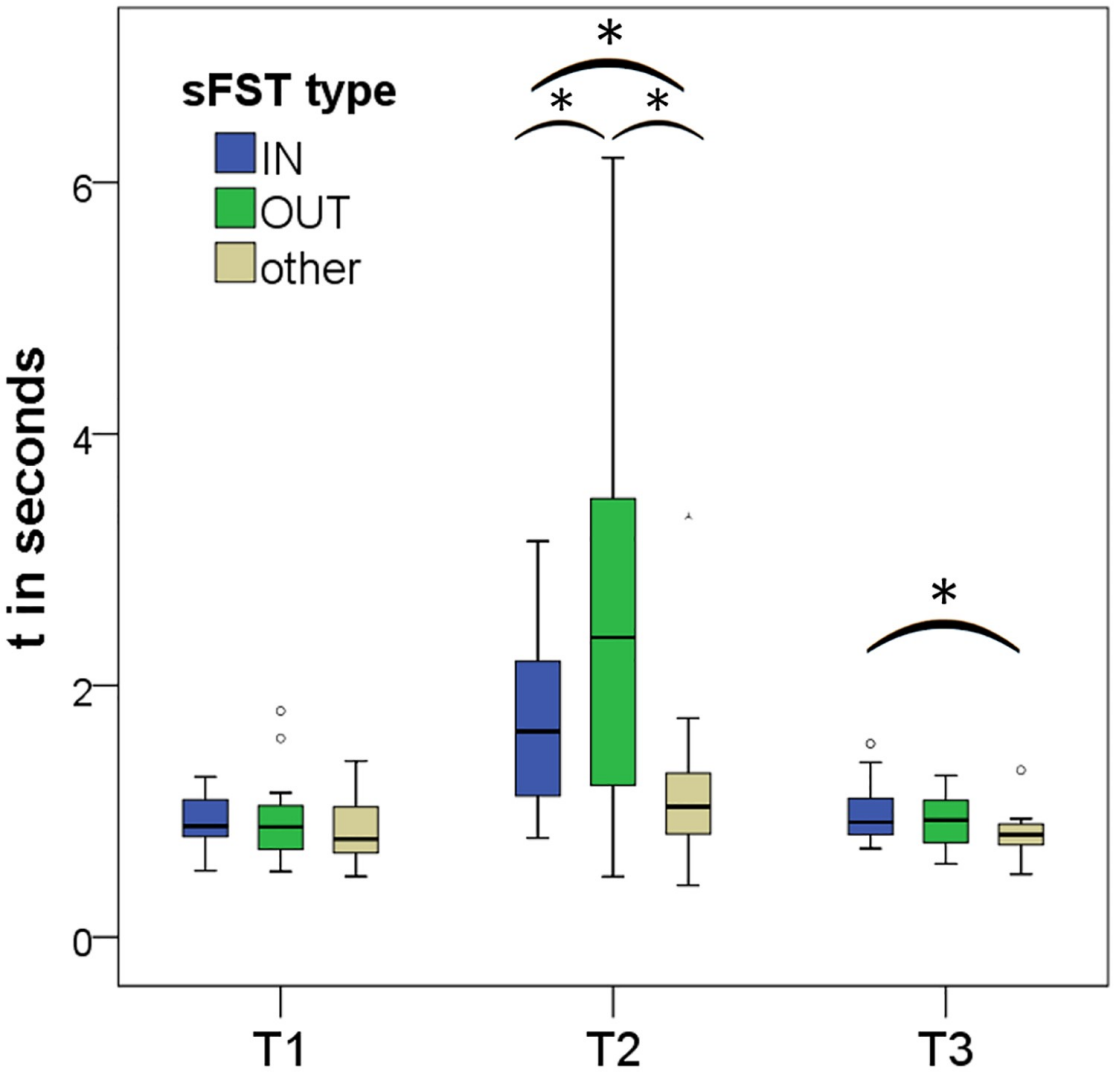
Boxplots of temporal aspects of spontaneous facial self-touch during (IN) and between sounds (OUT). The retention interval consisted of 40 sounds and 40 sound-free periods. Skin contact durations were the longest when facial self-touches were performed between sounds (OUT). T1 = movement time towards face; T2 = contact duration; T3 = movement time away from face; other = sFST during reproduction or exploration; *p < .05.

Post-hoc comparisons showed that: contact durations between the sounds (OUT) were significantly longer than during sounds (IN vs OUT: t(18) = -2.801, p < .05, d = 0.64, Fig 6) and than during other phases of the experiment (OUT vs other: t(18) = 3.355, p < .005, d = 0.77). The shortest contact durations occurred during the other experimental phases (IN vs other: t(18) = 2.296, p < .05, d = 0.53).

Movement times away from the face were significantly longer during sounds than during other phases of the experiment (IN vs other: t(18) = 2.473, p < .05; Table 2). All other movement times were statistically equal (IN vs OUT: t(18) = 1.181, p = .253; OUT vs other: t(18) = 1.654, p = .115).

### Laterality

#### Hypothesis 2a—No difference in hand use frequency

Across all experimental phases right-handed sFST (n = 164) were performed more often than left- handed sFST (n = 130) but the difference was only marginally significant (Binomial Test: p = .054; Table 3).

**Table 3:** Frequencies of sFST performed with either one or both hands.

experimental phase	right hand	left hand	both
exploration	19	12	2
retention interval (RI)	99	92	3
reproduction	46	26	4
total	164	130	9

Mean frequency of within-subjects left and right hand use (across all experimental phases, n = 54) was equal (right hand M = 3.84, SD = 3.11; left hand M = 3.47, SD = 2.58; Paired-samples t-test: t = 0.558, p = .581).

For those participants who performed sFST with both the right and the left hand during RI, within-subjects analyses revealed that both hands were used with the same mean frequency (left hand: M = 2.43, SD = 1.59; right hand: M = 3.09, SD = 2.27; Paired-samples t-test: t = 1.35, df = 22, p = .189). Test subjects who performed sFST with both the right and the left hand during reproduction (n = 6) also used both hands with the same frequency (right hand: M = 1.33, SD = 0.82; left hand: M = 1.83, SD = 1.33; Paired-samples t-test: t = -0.69, df = 5, p = .518).

#### Hypothesis 2b—Temporal aspects of hand use

Within-subject comparisons of those participants who performed sFST with both the right and the left hand movement times and contact durations of left- and right-handed sFST were the same during RI (n = 23; for paired-samples t-tests, means and SD see S1 Table) and during reproduction (n = 6; see S2 Table).

#### Hypothesis 3a—Effects of face area

**Fig 7:**
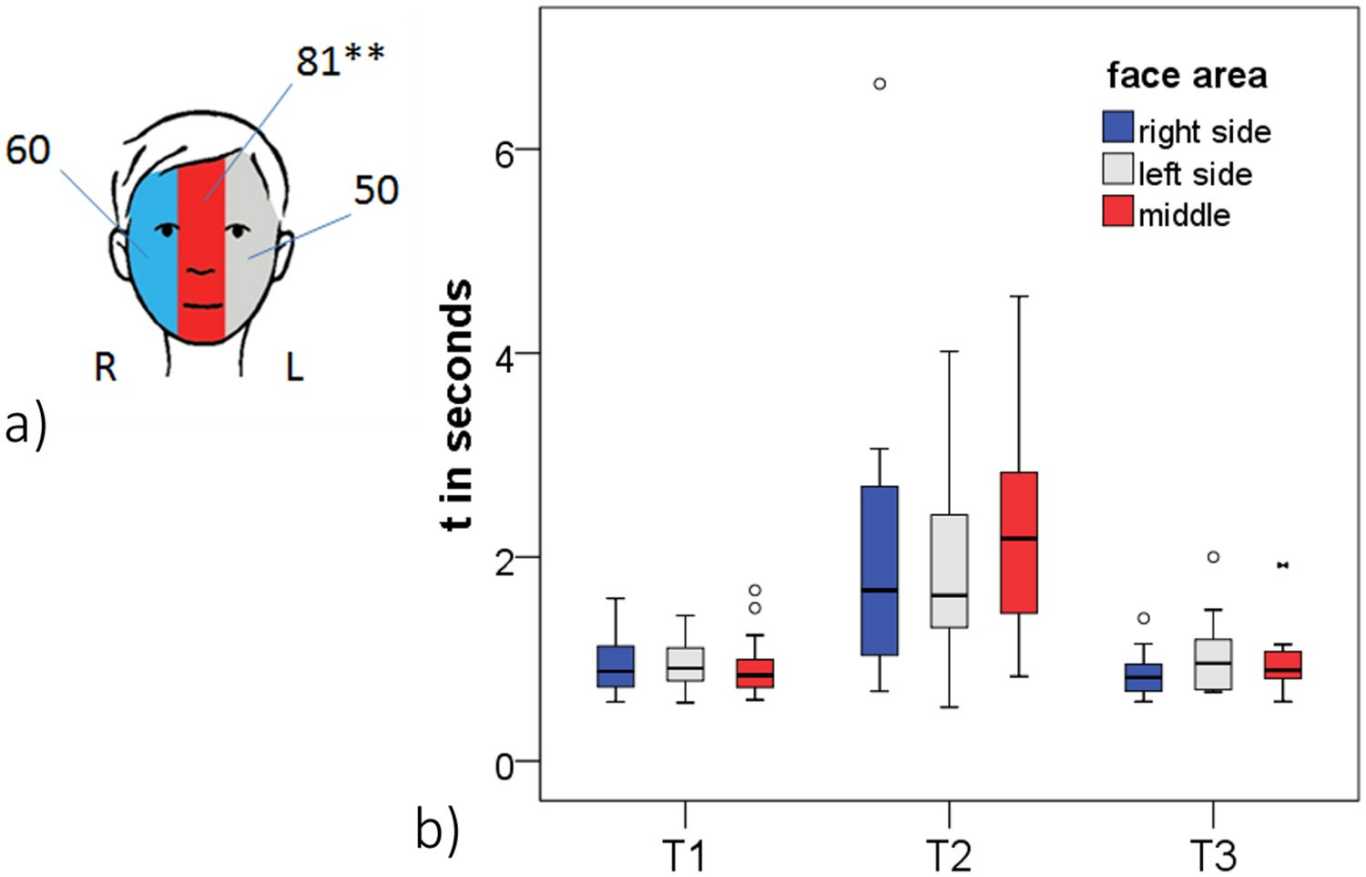
Self-touch of different face areas during the retention interval. a) Frequency of sFST per face area. b) Temporal aspects of sFST of different face areas. **p < .01; T1: movement time towards the face; T2: contact duration; T3: movement time away from face. (Face-graphic by C. Maiwald).

Frequency: Of the n = 45 participants who performed sFST during RI significantly most sFST were directed towards the middle axis of the face (middle vs. left side: p < .01; middle vs. right side: (p = .092); Fig 7a). The frequency of sFST towards the right and the left side of the face was equal (p = .391).

The differences between face areas during reproduction (n = 20 subjects; right = 25; middle = 28; left = 19) did not reach significance (left-side/right-side: p = .451; left-side/middle: p = .243; right-side/middle: p = .784). Neither did the differences in frequency during exploration (n = 18 subjects; right = 6; middle = 12; left = 13; left-side/right-side: p = .167; left-side/middle: p = 1.0; right-side/middle: p = .238).

Temporal aspects: For those participants who directed sFST to all three face areas (left, right, middle) during the retention interval (n = 14) one-way within-subjects ANOVAs of face area on hand movement times and contact durations were conducted. None of the comparisons reached significance (T1: F(2,12) = 0.041, p = .960; T2: F(2,12) = 0.324, p = .729; T3 = F(2,12) = 1.198, p = .335; Fig 7b), indicating that contact durations and movement times were the same for all three face areas.

Unfortunately, during exploration and reproduction too few subjects performed sFST in all three face areas to allow differentiated analyses.

#### Hypothesis 3b—Cumulative effects of hand and face-side

**Fig 8:**
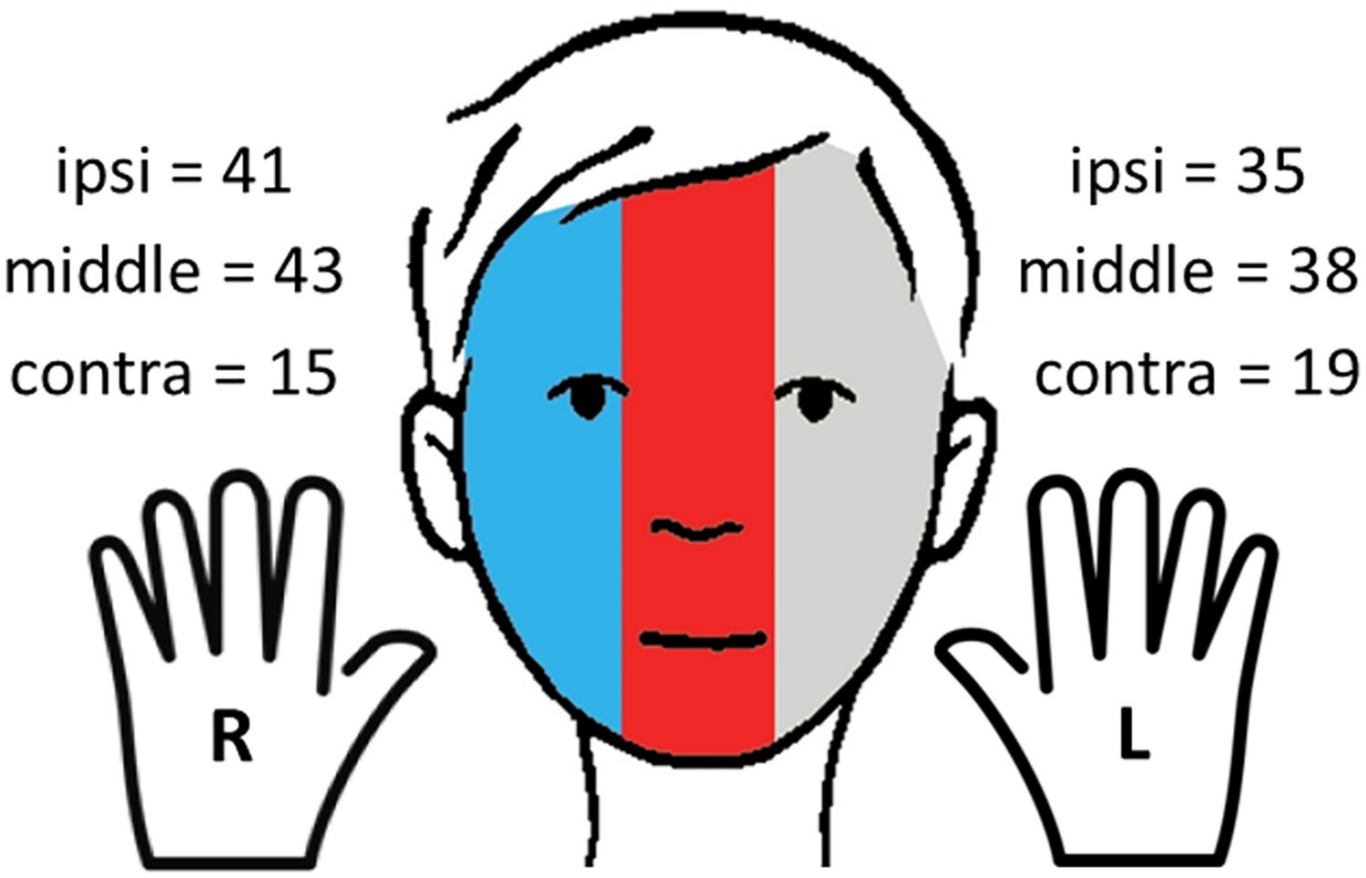
uency of sFST during the retention interval: Both hands were used equally often for ipsi- and contralateral self-touches. Ipsilateral self-touches occurred significantly more often than contralateral sFST. R = right hand, L = left hand; ipsi = ipsilateral side of face; m = middle axis; contra = contralateral side. (Hand-Icon made by Zlatko Najdenovski from flaticon.com; Face by C. Maiwald).

Frequency: Across the n = 45 participants who performed sFST during RI the right and the left hand were used equally often to touch the respective ipsilateral side of the face (right hand ipsi/ left hand ipsi: p = .567) and the middle axis (right hand middle/ left hand middle: p = .657; Fig 8). For both hands contralateral sFST were significantly less frequent than ipsilateral (left hand: p < .05; right hand: p < .001) or middle axis sFST (left hand: p < .05; right hand: p < .001). Only approximately a third of all sFST were directed to either contralateral side of the face.

Temporal aspects: Since no participant performed all six of the possible hand-face-combinations (right hand ipsilateral, right hand contralateral, right hand middle axis, left hand ipsilateral, left hand contralateral, left hand middle axis) within-subjects analyses with all variables were out of the question. Instead we decided to compare all possible combinations separately and (in case of significance) adjust alpha for multiple comparisons. However, none of the 15 possible combinations differed in mean movement time or mean contact duration (See S3 Table for paired samples t-tests of all possible hand-face area combinations.): Movements times and contact durations were the same for both hands in all analyzed areas of the face.

### Contact duration and point of touch

Since the most salient differences in sFST frequency and temporal aspects were found during (IN) and between sounds (OUT), we were curious whether any effects of hand or face side may be observable here. Explorative analyses across all subjects who performed sFST during RI (n = 54) were conducted.

#### Frequencies

The same relative frequencies of right and left-handed sFST were observed during and between sounds (Table 4). The face areas did, however, differ in the amount of sFST directed towards them (Table 4): by far the most sFST were directed towards the middle axis of the face during sounds. During silences all three face areas were touched with the same frequency.

**Table 4:** Frequencies of sFST per hand and face area during (IN) and between (OUT) sounds.

		sound	
		IN	OUT
**Hand used**	**right hand**	66	33
	**left hand**	63	28
	**both**	2	1
Face area*	**right side**	37	23
	**left side**	29	20
	**middle**	63	18

*bimanual sFST were excluded (n = 3)

#### Temporal aspects of *hand used* and sound

Two-way analyses of variance were conducted to compare the influence of ‘hand used’ (left, right) and ‘sound’ (IN, OUT) on the three temporal aspects of sFST (T1, T2, T3).

For T1 none of the effects were significant (sound: F(1,186) = .000, p = .983; hand used: F(1,186) = 2.646, p = .105; interaction: F(1,186) = .126, p = .723).

For T2 (contact duration) there was a significant main effect of sound (F(1,186) = 11.128, p < .001, Eta^2^ = .056) with longer sFST contact durations during silences–replicating the results shown under Hypothesis 1c by within-subject comparisons. Hand used (F(1,186) = .220, p = .640) and the interaction effect (F(1,184) = .060, p = .806) did not reach significance, however.

For T3 the main effect of hand used reached significance (F(1,186) = 4,820, p < .05, Eta^2^ = .025) with longer movement times away from the face for the left hand. The other effects did not reach significance (sound: F(1,186) = .935, p = .335; interaction: F(1,186) = 1,508, p = .221). Means and standard deviations are presented in S4 Table.

#### Temporal aspects of *face area* and sound

**Fig 9:**
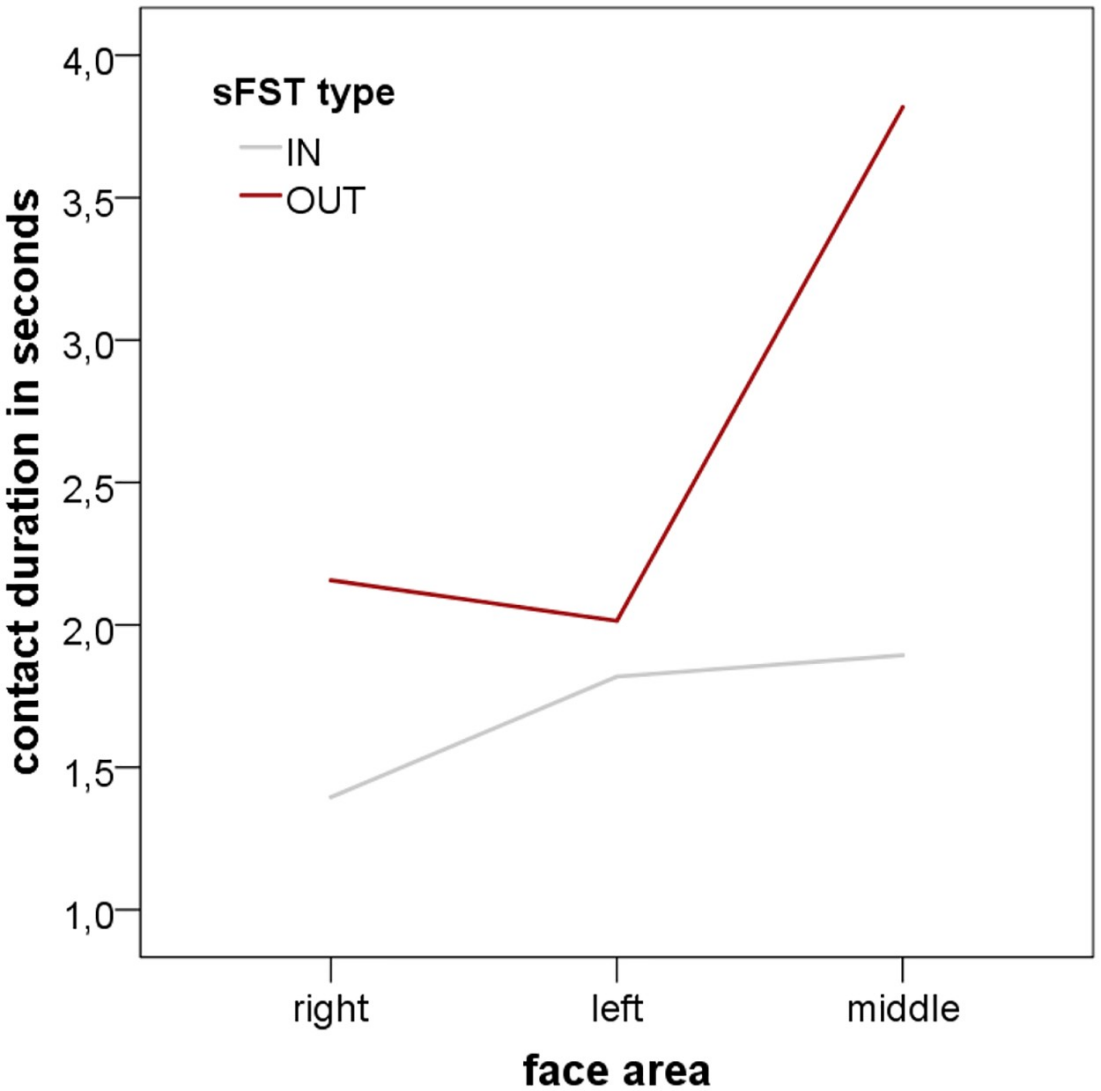
Two-way analysis of variance of face area and sound on contact duration. IN = sFST during sounds; OUT = sFST between sounds; The interaction effect and both main effects were significant: contact duration was the longest in the middle axis of the face during silences between the sounds.

Two-way analyses of variance were conducted to compare the influence of ‘face area’ (left, right, middle) and ‘sound’ (IN, OUT) on the three temporal aspects of sFST (T1, T2, T3). For T1 and T3 none of the effects were significant (T1: sound: F(1,184) = ,030, p = .862; face area: F(2,184) = ,458, p = .633; interaction: F(2,184) = ,201, p = .818; T3: sound: F(1,184) = ,888, p = .347; face area: F(2,184) = 1,709, p = .184; interaction: F(2,184) = 1,035, p = .357). But, for T2 (contact duration), both main effects (sound: F(1,184) = 14,236, p < .001, Eta^2^ = .072; face area: F(2,184) = 7,391, p < .001, Eta^2^ = .074) and the interaction effect (F(2,184) = 3,965, p < .05, Eta^2^ = .041) became significant (Fig 9), indicating significant differences in contact duration of the different face areas for IN and OUT. Corresponding means and standard deviations are presented in S5 Table.

## Discussion

The data for the present study were gathered in an experiment investigating discrete spontaneous facial self-touches (sFST) during a delayed memory task of complex haptic stimuli (sunken reliefs). For the first time in the history of self-touch research, the present experimental setting was designed to allow objective measurements of the movement and contact times of self-touch processes. Our aim was to investigate the temporal aspects of discrete facial self-touch with focus on the executing hand and touched face area.

On average participants performed M = 5.61 (SD = 4.81) sFST during the course of the experiment. This number is similar to the mean found by a previous study that used the same experimental setting [6]. Across all sFST that occurred during the experiment, hand movement towards and away from the face took approximately one second. The mean contact duration of all occurring sFST was 1.76 seconds (SD = 1.53), which is in line with the notion, that discrete ST are usually shorter than 3 seconds [14]. As the small variance indicates, sFST were very similar in duration and movement times and are, therefore, well distinguishable from continuous forms of ST.

Our results support previous findings that self touch frequency increases when attention is distracted and needs to be refocused. However, counterintuitively, frequency and duration of self-touch seem to be unrelated: we observed increases in frequency that were unrelated to the duration of self-touch as well as increases of contact duration while frequency remained low.

Concerning hand and face laterality we found that both hands were used equally often and with the same overall movement times and contact durations. However, we did find significant effects for face area in both frequency and contact durations. Therefore, this study showed, for the first time, that the point of touch has some relevance of its own, independently of which hand is used to perform the self-touch movement.

All results will be discussed in detail below.

### sFST frequency and duration increase when attention is distracted

In line with existing literature Hypothesis 1a was confirmed: During the retention interval significantly more sFST occurred than during all other experimental phases. This result mirrors the result found by Grunwald and colleagues [6] and supports the distraction susceptibility and attentional focusing hypothesis of Barroso and colleagues [12,2,13] stating that ST will increase when attention is distracted.

Barroso et al also stated that continuous concentration would warrant longer ST durations [12]. In line with this, within-subjects comparisons revealed that movement times and contact durations were significantly longer during RI than during the pooled other experimental phases (Hypothesis 1b). Even though participants had to concentrate during exploration and reproduction as well, they were not forced to sustain concentration. Since no time limit was set for these periods, they were free to start over if they felt the need. Contrary to that, during RI do-overs were not possible and continuous concentration was warranted to avoid losing the working memory contents.

To take the analyses one step further, we also investigated whether sFST would occur predominantly during distracting sounds or during silences. Significantly more sFST occurred during the acoustic stimuli (IN) than during the silences between the sounds (OUT; Hypothesis 1c) corroborating the attention capture potential of changing-state acoustic stimuli [27,24,40,41,26,25].

We also found that sFST that occurred during the silences between the sounds (OUT) lasted significantly longer than sFST during the sounds. This inexpected result may be caused by an anticipation effect. The participants may have braced themselves to concentrate on their memory contents and not to get distracted by the next sound, but this anticipation may have been a distraction from the memory contents in itself: Studies investigating the effects of aversive stimuli during anticipation settings showed that the antecedent psychophysiological effects of these stimuli are stronger when they were anticipated than when they occur unexpectedly [42,43]. Therefore, anticipating the next sound may have influenced the psychophysiological state of the participants and thereby disturbed the necessary concentration for working memory rehearsal and refreshing [44].

During the present analyses the associations between frequency and temporal aspects were ambiguous. We observed increases in frequency that were unrelated to the duration of self-touch as well as increases of contact duration while frequency remained low. The mechanisms behind these effects are currently unknown. Future studies should try to investigate the association of psychophysiological state and duration of sFST more thoroughly.

### Left- and right-handed sFST

Researchers have studied the laterality of ST based on the assumption, that the muscular and sensory activity of the ST movements may be associated with the cognitive and emotional activation of the contralateral brain hemisphere and its straying activation to adjacent brain areas [3]. Based on previous findings we expected to observe more left-handed sFST in the course of the present experiment (Hypothesis 2a). However, right- and left-handed sFST were performed equally often during all experimental phases. In line with Ruggieri et al (1982) we also expected movement times and contact durations to be significantly longer for left-handed ST than for right-handed ST (Hypothesis 2b). Within-subjects comparisons of movement times and contact durations did not reveal any significant differences between left- and right-handed sFST, except a tendency for slightly longer movement times of left-handed sFST. However, since our setting did not elicit the expected amplification of left-handed sFST we need to bring into question why that was not the case. Previous studies that elicited higher left-handed ST frequencies have either investigated patients with depressive symptoms, anxiety or high field dependence [30,31] or healthy subjects had to answer embarrassing questions [32,33]. While depression, anxiety and embarrassment all constitute negative emotional states they may still be very different from the states that were induced by the sounds that were used in the present study. It is possible that the sounds we used were more irritating than sad or anxiety-provoking and, therefore, triggered a different pattern of sFST. Future investigations should try to utilize sounds of a more homogenous affective nature.

### Most and longest ST occurred in the middle axis of the face

To the best of our knowledge all previous experiments investigating ST and laterality have focused on the executing hand but did not take into account which side of the body or of the face was touched. If, however, the skin contact aspect of sFST is of central relevance in the sFST process, this should be reflected in the frequency and contact durations of the different face areas (Hypothesis 3a). First, preference of face area was analyzed across all sFST of the retention interval: We found that most sFST were directed to the middle axis of the face. However, we did not find any significant differences in movement times or contact durations between the face areas, even though contact duration on the middle axis of the face was descriptively the longest of the three face areas. Also, there were no cumulative effects in the temporal aspects of hand and face-side (Hypothesis 3b).

Since the most salient differences in sFST times and frequency were found during and between sounds, we were curious whether any effects of hand or face side may be observable here. Again, analyses revealed that both hands were used equally often during sounds (IN) and during silences (OUT). But the analysis of the face areas revealed that a significant majority of sFST was directed towards the middle axis of the face during sounds while all three face areas were touched equally often during silences. Movement times did not differ between face areas or IN and OUT. The analyses of contact durations, however, revealed both a significant interaction effect and main effects: contact durations were significantly longer during silences than during sounds with the significantly longest mean contact duration in the middle of the face during silences. In other words, while both hands were used equally often and with the same overall movement times and contact durations, we did find significant effects for face area in both frequency and contact durations. Ergo the point of touch seems to have some relevance of its own, independently of which hand is used to perform it.

**Fig 10:**
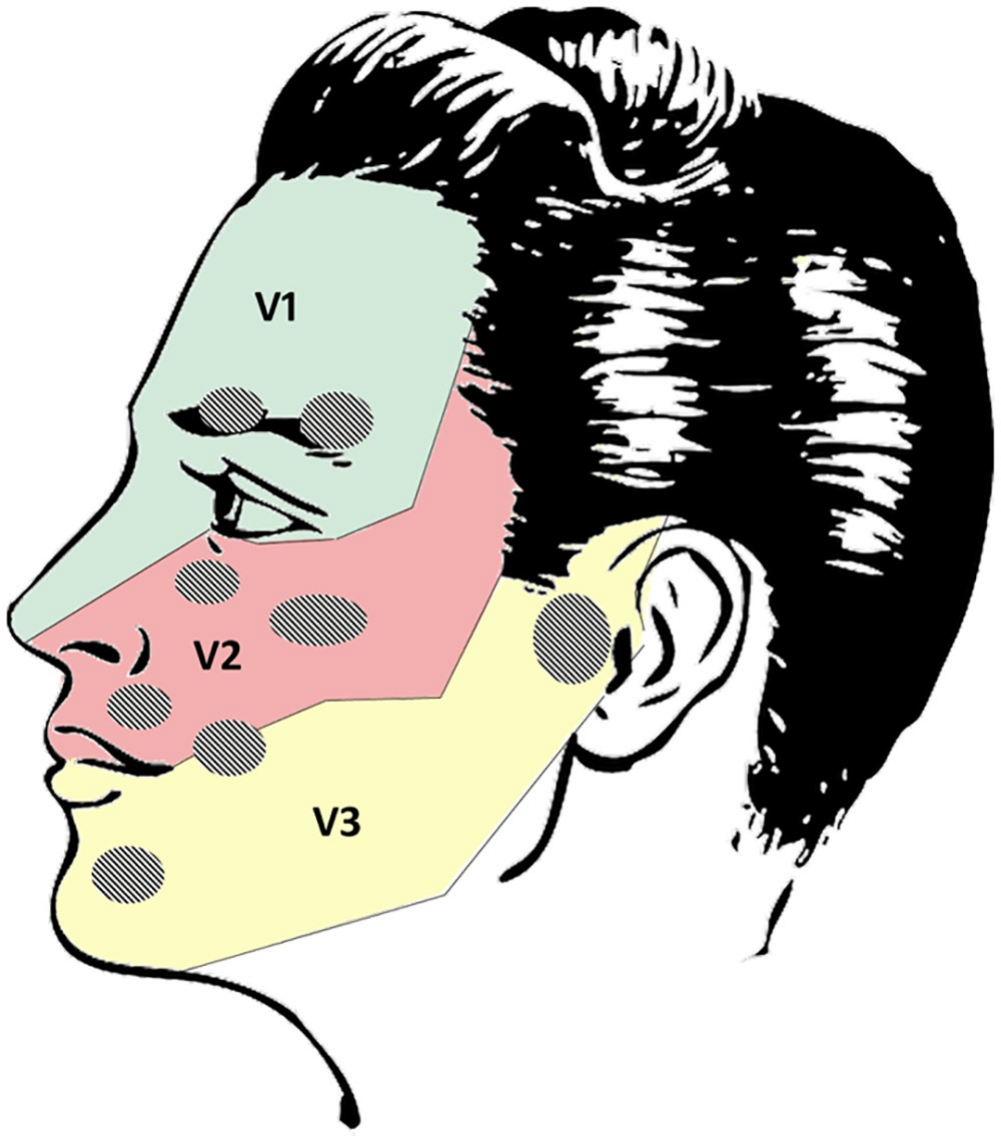
Sensory innervation areas of trigeminal nerve and areas of communicating rami of facial and trigeminal nerve. Innervation areas: V1 = ophthalmic; V2 = maxillary; V3 = mandibular branch of nervus trigeminus; gray dots: connections of superficial branches of trigeminal nerve and facial nerve [47] (Face by Clker-Free-Vector-Images).

Since we were interested in investigating differences in laterality, subdividing the face into longitudinal areas seemed appropriate. However, other clustering possibilities are conceivable: a) The face could be subdivided into areas of high and low sensitivity. Sensitivity thresholds of the face have been reported to decrease from the forehead to the midface, followed by the chin and the lip [45], with the highest concentration of facial mechanoreceptors at the corners of the mouth [46,47]. b) Facial areas could also be clustered according to the sensory fields of the fifth cranial nerve (Nervus trigeminus; [48] (Fig 10). As opposed to right- or left-sided self-touch, middle axis sFST may be registered by trigeminal nerve branches of both face sides and may, therefore, be processed by both hemispheres. Future studies should investigate whether middle-axis sFST are directed predominantly to the mouth, nose, chin or eyes. Skin areas above peripheral facial and trigeminal nerve communications should also be considered as special points of interest (Fig 10). In current literature there is no common agreement on the function of these communicating rami [47]. An involvement with facial muscle proprioception seems to be most likely [49]. Anecdotally there seems to be an overlap of these anatomical sites and those areas where sFST are most often directed: the eyebrows, infra-orbital area close to the nose, and the corners of the mouth.

As previous results have shown that sFST coincide with EEG changes [6] future research should investigate if sFST of different face areas differ in their EEG activation.

## Conclusion

Currently, it is much too early for a conclusive theory of self-touch. Despite decades of research we know very little about the physiological fundamentals of this behavior. In the present study we were able to show that cognitive and emotional load play a role in the occurrence of self-touch directed towards the face. But why are sFST directed towards varying face areas? And what triggers these movements? Is it possible that self-touches are only an epiphenomenon of a much more sophisticated neuronal mechanism? It is conceivable that immediately prior to sFST a faint sensory signal may be sent to the skin without any external dermal stimulation. Top-down excitation of dermal receptors causing skin sensations is known through phenomena like contagious itch [50] or somatic hallucinations [51]. If this is the case it would support the notion that the point of touch is at least as relevant to the self-touch process as the executing hand. And it would welcome an entirely new approach to this omnipresent phenomenon.

## Supporting information

S1 Table Within-subjects comparisons of movement times and contact duration *during RI*. T1 = movement time towards face; T2 = sFST skin contact duration; T3 = movement time away from face; Paired-samples t-tests of movement times and contact durations for left- and right-handed sFST indicated no significant differences.
(PDF)

S2 Table Within-subjects comparisons of movement times and contact duration *during reproduction*. T1 = movement time towards face; T2 = sFST skin contact duration; T3 = movement time away from face; Subjects who performed both left- and right-handed sFST during reproduction (n = 6) did not differ in the temporal aspects of their left- and right-handed sFST (paired-samples t-tests).
(PDF)

S3 Table Paired samples t-Tests of all possible hand-face area combinations of sFST. T1 = movement time towards face; T2 = contact duration; T3 = movement time away from face; R = right hand; L = left hand; ipsi = ipsilateral face area; con = contralateral face area; med = middle axis of the face. For each hand-face-area combination only those subjects were compared who performed both types of sFST. By way of example, the paired-samples t-test of Pair 1 only includes subjects who performed both ipsi- and contralateral sFST with their right hand (n = 13).
(PDF)

S4 Table Means and SD of temporal aspects in seconds for hand used and sound. IN = during sounds; OUT = between sounds.
(DOCX)

S5 Table Means and SD of temporal aspects in seconds for face area and sound. IN = during sounds; OUT = between sounds.
(DOCX)
