# Supplementary material for: Self-touch: Contact durations and point of touch of spontaneous facial self-touches differ depending on cognitive and emotional load
Source: PLoS One. 2019 Mar 12;14(3):e0213677. doi: 10.1371/journal.pone.0213677 (PMC6413902; doi:10.1371/journal.pone.0213677)
Supplement: S1 Table — T1 = movement time towards face; T2 = sFST skin contact duration; T3 = movement time away from face; Paired-samples t-tests of movement times and contact durations for left- and right-handed sFST indicated no significant differences. (PDF) [file pone.0213677.s001.pdf]

Table S1. Within-subjects comparisons of movement times and contact duration *during RI*

temporal aspects		*M*	*N*	*SD*	*t*	*df*	*p*
Pair 1	T1 right hand	.944	23	.300	.588	22	.563
	T1 left hand	.909	23	.229			
Pair 2	T2 right contact	1.938	23	1.376	.222	22	.826
	T2 left contact	1.832	23	1.722			
Pair 3	T3 right hand	.983	23	.247	-.980	22	.338
	T3 left hand	1.326	23	1.668			

T1 = movement time towards face; T2 = sFST skin contact duration; T3 = movement time away from face; Paired-samples t-tests of movement times and contact durations for left- and right-handed sFST indicated no significant differences.
