# Supplementary material for: Self-touch: Contact durations and point of touch of spontaneous facial self-touches differ depending on cognitive and emotional load
Source: PLoS One. 2019 Mar 12;14(3):e0213677. doi: 10.1371/journal.pone.0213677 (PMC6413902; doi:10.1371/journal.pone.0213677)
Supplement: S2 Table — T1 = movement time towards face; T2 = sFST skin contact duration; T3 = movement time away from face; Subjects who performed both left- and right-handed sFST during reproduction (n = 6) did not differ in the temporal aspects of their left- and right-handed sFST (paired-samples t-tests). (PDF) [file pone.0213677.s002.pdf]

**Table S2. Within-subjects comparisons of movement times and contact duration *during reproduction***

| temporal aspects | | *M* | *N* | *SD* | *t* | *df* | *p* |
|---|---|---|---|---|---|---|---|
| T1 | right hand | .6044 | 6 | .18612 | -.800 | 5 | .460 |
| | left hand | .7023 | 6 | .33855 | | | |
| T2 | right contact | .8542 | 6 | .38802 | -1.555 | 5 | .181 |
| | left contact | 1.2384 | 6 | .62103 | | | |
| T3 | right hand | .6059 | 6 | .11644 | -.987 | 5 | .369 |
| | left hand | .8180 | 6 | .46650 | | | |

T1 = movement time towards face; T2 = sFST skin contact duration; T3 = movement time away from face; Subjects who performed both left- and right-handed sFST during reproduction (n = 6) did not differ in the temporal aspects of their left- and right-handed sFST (paired-samples t-tests).
