# Supplementary material for: Self-touch: Contact durations and point of touch of spontaneous facial self-touches differ depending on cognitive and emotional load
Source: PLoS One. 2019 Mar 12;14(3):e0213677. doi: 10.1371/journal.pone.0213677 (PMC6413902; doi:10.1371/journal.pone.0213677)
Supplement: S3 Table — T1 = movement time towards face; T2 = contact duration; T3 = movement time away from face; R = right hand; L = left hand; ipsi = ipsilateral face area; con = contralateral face area; med = middle axis of the face. For each hand-face-area combination only those subjects were compared who performed both types of sFST. By way of example, the paired-samples t-test of Pair 1 only includes subjects who performed both ipsi- and contralateral sFST with their right hand (n = 13). (PDF) [file pone.0213677.s003.pdf]

S3 Table. Paired samples t-Tests of all possible hand-face area combinations of sFST.

		M	*N*	*SD*	*t*	*df*	*p*
Pair 1	T1_R_ipsi	.916	13	.309	.836	12	.419
	T1_R_con	.856	13	.301			
	T2_R_ipsi	1.476	13	.817	1.413	12	.183
	T2_R_con	1.119	13	.541			
	T3_R_ipsi	.920	13	.247	1.522	12	.154
	T3_R_con	.851	13	.206			
Pair 2	T1_L_ipsi	.949	12	.210	1.210	11	.252
	T1_L_con	.837	12	.205			
	T2_L_ipsi	1.306	12	.694	-1.185	11	.261
	T2_L_con	1.537	12	.674			
	T3_L_ipsi	.980	12	.379	.580	11	.573
	T3_L_con	.889	12	.258			
Pair 3	T1_R_con	.934	9	.347	.563	8	.589
	T1_L_con	.853	9	.214			
	T2_R_con	1.524	9	1.067	-.474	8	.648
	T2_L_con	1.669	9	.691			
	T3_R_con	1.055	9	.194	1.685	8	.131
	T3_L_con	.893	9	.234			
Pair 4	T1_R_ipsi	.872	18	.248	-1.161	17	.262
	T1_L_ipsi	.950	18	.221			
	T2_R_ipsi	1.517	18	1.381	-.284	17	.780
	T2_L_ipsi	1.620	18	.988			
	T3_R_ipsi	.876	18	.241	-1.775	17	.094
	T3_L_ipsi	1.051	18	.338			
Pair 5	T1_R_ipsi	.817	11	.235	-.746	10	.473
	T1_L_con	.913	11	.311			
	T2_R_ipsi	1.140	11	.397	-.962	10	.359
	T2_L_con	1.360	11	.708			
	T3_R_ipsi	.884	11	.232	-.450	10	.662
	T3_L_con	.922	11	.290			

Pair 6	T1_L_ipsi	.934	13	.249	-.363	12	.723
	T1_R_con	.976	13	.413			
	T2_L_ipsi	1.607	13	.900	.532	12	.604
	T2_R_con	1.417	13	.893			
	T3_L_ipsi	.980	13	.403	.007	12	.995
	T3_R_con	.979	13	.232			
Pair 7	T1_R_med	.907	15	.354	-.024	14	.981
	T1_L_med	.911	15	.425			
	T2_R_med	1.783	15	.980	.481	14	.638
	T2_L_med	1.603	15	1.037			
	T3_R_med	.908	15	.343	-.055	14	.957
	T3_L_med	.914	15	.258			
Pair 8	T1_R_med	.860	17	.326	-.754	16	.462
	T1_R_ipsi	.937	17	.297			
	T2_R_med	1.824	17	1.062	.335	16	.742
	T2_R_ipsi	1.688	17	1.466			
	T3_R_med	.903	17	.329	.540	16	.596
	T3_R_ipsi	.854	17	.211			
Pair 9	T1_R_med	.818	16	.263	-1.449	15	.168
	T1_R_con	.984	16	.403			
	T2_R_med	1.956	16	1.024	1.547	15	.143
	T2_R_con	1.360	16	.910			
	T3_R_med	.835	16	.198	-1.005	15	.331
	T3_R_con	.918	16	.217			
Pair 10	T1_R_med	.885	18	.338	-.093	17	.927
	T1_L_ipsi	.893	18	.177			
	T2_R_med	1.839	18	1.001	.655	17	.521
	T2_L_ipsi	1.638	18	.859			
	T3_R_med	.980	18	.402	.243	17	.811
	T3_L_ipsi	.952	18	.344			
Pair 11	T1_R_med	.789	8	.213	-1.832	7	.110
	T1_L_con	.891	8	.194			

	T2_R_med	1.767	8	1.176	.109	7	.916
	T2_L_con	1.700	8	.732			
	T3_R_med	.822	8	.182	-.426	7	.683
	T3_L_con	.853	8	.214			
Pair 12	T1_L_med	.887	18	.271	-.453	17	.656
	T1_R_ipsi	.921	18	.325			
	T2_L_med	1.774	18	1.946	.338	17	.739
	T2_R_ipsi	1.590	18	1.447			
	T3_L_med	1.416	18	1.878	1.344	17	.197
	T3_R_ipsi	.838	18	.213			
Pair 13	T1_L_med	.917	11	.495	.827	10	.428
	T1_R_con	.834	11	.424			
	T2_L_med	2.422	11	2.450	1.333	10	.212
	T2_R_con	1.364	11	.802			
	T3_L_med	1.621	11	2.423	.977	10	.352
	T3_R_con	.893	11	.245			
Pair 14	T1_L_med	.866	20	.405	-.956	19	.351
	T1_L_ipsi	.955	20	.274			
	T2_L_med	1.860	20	1.874	-.106	19	.917
	T2_L_ipsi	1.913	20	.968			
	T3_L_med	1.356	20	1.796	.554	19	.586
	T3_L_ipsi	1.138	20	.366			
Pair 15	T1_L_med	.872	8	.259	-.158	7	.879
	T1_L_con	.897	8	.240			
	T2_L_med	1.641	8	.606	-.539	7	.606
	T2_L_con	1.791	8	.599			
	T3_L_med	.959	8	.245	.291	7	.779
	T3_L_con	.925	8	.330			

T1 = movement time towards face; T2 = contact duration; T3 = movement time away from face; R = right hand; L = left hand; ipsi = ipsilateral face area; con = contralateral face area; med = middle axis of the face. For each hand-face-area combination only those subjects were compared who performed both types of sFST. By way of example, the paired-samples t-test of Pair 1 only includes subjects who performed both ipsi- and contralateral sFST with their right hand (n = 13).
