# Supplementary material for: Self-touch: Contact durations and point of touch of spontaneous facial self-touches differ depending on cognitive and emotional load
Source: PLoS One. 2019 Mar 12;14(3):e0213677. doi: 10.1371/journal.pone.0213677 (PMC6413902; doi:10.1371/journal.pone.0213677)
Supplement: S4 Table — IN = during sounds; OUT = between sounds. (DOCX) [file pone.0213677.s004.docx]

**S4 Table. Means and SD of temporal aspects in seconds for hand used and sound.**

| **T1 (movement towards face)** | | | | | |
| --- | --- | --- | --- | --- | --- |
| **sound** | **Hand used** | **M** | | **SD** | **N (sFST)** |
| IN | right | .91776 | | .372466 | 66 |
|  | left | 1.02425 | | .283620 | 63 |
|  | total | .96976 | | .335064 | 129 |
| OUT | right | .93572 | | .346322 | 33 |
|  | left | 1.00405 | | .398547 | 28 |
|  | total | .96709 | | .369627 | 61 |
| Total | right | .92375 | | .362287 | 99 |
|  | left | 1.01803 | | .321176 | 91 |
|  | total | .96890 | | .345554 | 190 |
| **T2 (contact duration)** | | | | | |
| IN | right | 1.82433 | 1.303599 | | 66 |
|  | left | 1.63889 | 1.163962 | | 63 |
|  | total | 1.73377 | 1.236064 | | 129 |
| OUT | right | 2.62654 | 2.271481 | | 33 |
|  | left | 2.56864 | 2.412179 | | 28 |
|  | total | 2.59996 | 2.317546 | | 61 |
| Total | right | 2.09173 | 1.719412 | | 99 |
|  | left | 1.92497 | 1.692654 | | 91 |
|  | total | 2.01186 | 1.704182 | | 190 |
| **T3 (movement away from face)** | | | | | |
| IN | right | .97248 | .472300 | | 66 |
|  | left | 1.07578 | .353229 | | 63 |
|  | total | 1.02293 | .420000 | | 129 |
| OUT | right | .94460 | .332158 | | 33 |
|  | left | 1.31013 | 1.511023 | | 28 |
|  | total | 1.11238 | 1.058305 | | 61 |
| Total | right | .96319 | .429131 | | 99 |
|  | left | 1.14789 | .884725 | | 91 |
|  | total | 1.05165 | .690491 | | 190 |

IN = during sounds; OUT = between sounds
