# Supplementary material for: Self-touch: Contact durations and point of touch of spontaneous facial self-touches differ depending on cognitive and emotional load
Source: PLoS One. 2019 Mar 12;14(3):e0213677. doi: 10.1371/journal.pone.0213677 (PMC6413902; doi:10.1371/journal.pone.0213677)
Supplement: S5 Table — IN = during sounds; OUT = between sounds. (DOCX) [file pone.0213677.s005.docx]

S5 Table. Means and SD of temporal aspects in seconds for face area and sound.

| **T1 (movement towards face)** | | | | | |
| --- | --- | --- | --- | --- | --- |
| **sound** | **Face area** | **M** | | **SD** | **N (sFST)** |
| IN | right side | .97746 | | .302781 | 37 |
|  | left side | .98141 | | .255333 | 29 |
|  | middle | .95989 | | .385884 | 63 |
|  | total | .96976 | | .335064 | 129 |
| OUT | right side | 1.00085 | | .352081 | 23 |
|  | left side | .98594 | | .366956 | 20 |
|  | middle | .90299 | | .406169 | 18 |
|  | total | .96709 | | .369627 | 61 |
| Total | right side | .98642 | | .319832 | 60 |
|  | left side | .98326 | | .302220 | 49 |
|  | middle | .94724 | | .388620 | 81 |
|  | total | .96890 | | .345554 | 190 |
| **T2 (contact duration)** | | | | | |
| IN | right side | 1.39535 | .711871 | | 37 |
|  | left side | 1.81856 | 1.327680 | | 29 |
|  | middle | 1.89348 | 1.402260 | | 63 |
|  | total | 1.73377 | 1.236064 | | 129 |
| OUT | right side | 2.15676 | 1.374964 | | 23 |
|  | left side | 2.01406 | 2.098993 | | 20 |
|  | middle | 3.81727 | 3.057327 | | 18 |
|  | total | 2.59996 | 2.317546 | | 61 |
| Total | right side | 1.68723 | 1.074020 | | 60 |
|  | left side | 1.89836 | 1.667824 | | 49 |
|  | middle | 2.32099 | 2.039085 | | 81 |
|  | total | 2.01186 | 1.704182 | | 190 |
| **T3 (movement away from face)** | | | | | |
| IN | right side | .90963 | .267403 | | 37 |
|  | left side | 1.10481 | .390073 | | 29 |
|  | middle | 1.05178 | .492243 | | 63 |
|  | total | 1.02293 | .420000 | | 129 |
| OUT | right side | 1.01410 | .399865 | | 23 |
|  | left side | 1.01152 | .226011 | | 20 |
|  | middle | 1.35004 | 1.898452 | | 18 |
|  | total | 1.11238 | 1.058305 | | 61 |
| Total | right side | .94967 | .325383 | | 60 |
|  | left side | 1.06673 | .333352 | | 49 |
|  | middle | 1.11806 | .984494 | | 81 |
|  | total | 1.05165 | .690491 | | 190 |

IN = during sounds; OUT = between sounds
